# Methylone and Monoamine Transporters: Correlation with Toxicity

**DOI:** 10.2174/157015911795017425

**Published:** 2011-03

**Authors:** Chiharu Sogawa, Norio Sogawa, Kazumi Ohyama, Ruri Kikura-Hanajiri, Yukihiro Goda, Ichiro Sora, Shigeo Kitayama

**Affiliations:** a Department of Dental Pharmacology, Okayama University Graduate School of Medicine, Dentistry and Pharmaceutical Sciences, 2-5-1 Shikata-cho, Okayama 700-8525, Japan; b RI Research Center, Okayama University Dental School of Medicine, Dentistry and Pharmaceutical Sciences, 2-5-1 Shikata-cho, Okayama 700-8525, Japan; c Division of Pharmacognosy, Phytochemistry and Narcotics, National Institute of Health Sciences, 1-18-1 Kamiyoga, Setagaya-ku, Tokyo 158-8501, Japan; d Department of Biological Psychiatry, Tohoku University Graduate School of Medicine, 1-1 Seiryo-machi, Aoba-ku, Sendai 980-8574, Japan

**Keywords:** Methylone, neurotransmitter transporter, uptake, cocaine, methamphetamine, MDMA.

## Abstract

Methylone (2-methylamino-1-[3,4-methylenedioxyphenyl]propane-1-one) is a synthetic hallucinogenic amphetamine analog, like MDMA (3,4-methylenedioxy- methamphetamine), considered to act on monoaminergic systems. However, the psychopharmacological profile of its cytotoxicity as a consequence of monoaminergic deficits remains unclear. We examined here the effects of methylone on the transporters for dopamine (DAT), norepinephrine (NET), and serotonin (SERT), using a heterologous expression system in CHO cells, in association with its cytotoxicity. Methylone inhibited the activities of DAT, NET, and SERT, but not GABA transporter-1 (GAT1), in a concentration-dependent fashion with a rank order of NET > DAT > SERT. Methylone was less effective at inhibiting DAT and NET, but more effective against SERT, than was methamphetamine. Methylone alone was not toxic to cells except at high concentrations, but in combination with methamphetamine had a synergistic effect in CHO cells expressing the monoamine transporters but not in control CHO cells or cells expressing GAT1. The ability of methylone to inhibit monoamine transporter function, probably by acting as a transportable substrate, underlies the synergistic effect of methylone and methamphetamine.

## INTRODUCTION

Various illegal and/or restricted “designer drugs” have been created by modifying amphetamines. Methylone (2-methylamino-1-[3,4-methylenedioxy- phenyl]propane-1-one) is one such synthetic hallucinogenic amphetamine analogue, which resembles MDMA (3,4-methylenedioxymethamphetamine) but differs structurally by the presence of a ketone at the benzylic position [1, 2]. This compound has been newly placed under legal control as a drug of abuse in Japan [3]. 

Because of its structural similarity to MDMA, methylone is thought to act on monoaminergic systems. A behavioral study by Dal Cason *et al*. [4] found that methylone substituted for MDMA in rats trained to discriminate MDMA from saline. In studies of its pharmacology in vitro methylone was threefold less potent than MDMA at inhibiting platelet serotonin transporter (SERT) and as potent as MDMA in inhibiting transporters for dopamine (DAT) and norepinephrine (NET), but only weakly inhibited the vesicular monoamine transporter [5, 6]. However, there have been few pharmacological investigations of methylone in animal models, or studies about its mechanism of action. 

In addition, the cytotoxicity of MDMA is considered a consequence of monoaminergic deficits through the drug’s effects on the plasmalemmmal and vesicular monoamine transporters [7, 8]. Again, methylone may resemble MDMA in cytotoxic profiles. However, Nakagawa *et al*. [9] reported that methylone did not exhibit cytotoxic effects on isolated rat hepatocytes in contrast to MDMA and its analogues. The possibility can not be excluded that methylone is cytotoxic in some circumstances, since Nagai *et al*. [6] observed that like MDMA, it reduced mitochondrial membrane potential. 

Recently, we examined the effects of 5-methoxy-N, N-diisopropyltryptamine (5-MeO-DIPT), known as Foxy, on monoamine neurotransmitter transporters, including DAT, NET and SERT, using a heterologous expression system in COS-7 cells and rat brain synaptosomes, in association with its cytotoxicity [10]. In the present study, we used the same strategy to evaluate the relationship between the effects of methylone and methamphetamine on monoamine transporters and cell toxicity.

The results indicated an ability of methylone to inhibit monoamine transporter function, and cause damage synergistcally with methamphetamine in cells heterologously expressing monoamine transporters, suggesting that the transport of these drugs underlies their cytotoxicity. 

## MATERIALS AND METHODS

### Materials

Methylone was synthesized at the Division of Pharmacognosy, Phytochemistry and Narcotics, National Institute of Health Sciences (Tokyo, Japan). Its structure and purity were confirmed by melting point (degradation, 225°C), TLC, GC-MS and ^1^H-NMR analyses [11]. Other chemicals used were purchased from commercial sources. [^3^H]Dopamine (DA) (1.29 TBq/mmol), [^3^H]serotonin (5-HT) (1.04 TBq/mmol), and [^3^H]GABA (1.2 TBq/mmol) were obtained from PerkinElmer Life Science, Inc. (Boston, MA, USA), and [^3^H] norepinephrine (NE) (1.18 TBq/mmol) from GE healthcare Bioscience, Inc. (Buckinghamshire, UK). 

### Cell Culture and Expression

Chinese hamster ovary (CHO) cells were cultured at 37°C under 5 % CO_2 _/ 95 % air in Minimum Essential Medium-alpha (α-MEM) supplemented with 10 % fetal calf serum, 100 units/ml penicillin G, 100 μg/ml streptomycin, and 0.25 μg/ml fungisone. 

For the preparation of cell lines stably expressing transporters, CHO cells at subconfluence were transfected with cDNA of rat DAT (rDAT), NET (rNET), or SERT (rSERT), or mouse GABA transporter-1 (mGAT1) using FuGENE6 transfection reagent (Roche Diagnostics, Mannheim, Germany) according to the manufacturer’s directions. The cells were then diluted sequentially, seeded in 96-well plates, and selected using G418. The cell lines were confirmed to stably express the transporters based on the uptake of each tritium-labeled ligand, and designated CHO/rDAT, CHO/rNET, CHO/rSERT and CHO/mGAT1, respectively.

### Uptake and Release Assay

The uptake assay using radio-labeled ligands was performed, as described previously [10, 12]. Cells were washed three times with an oxygenated Krebs Ringer HEPES-buffered solution (KRH; 125 mM NaCl, 5.2 mM KCl, 1.2 mM CaCl_2_, 1.4 mM MgSO_4_, 1.2 mM KH_2_PO_4_, 5 mM glucose, and 20 mM HEPES, pH 7.3) and incubated for 10 min at 37°C with 10 nM of [^3^H]DA or other radio-labeled ligand. Nonspecific uptake was determined in mock-transfected cells and also in each plate in the presence of 100 μM cocaine for monoamine and 1 mM nipecotic acid for GABA. Data were analyzed using Eadie-Hofstee plots with Prism 5 (GraphPad Software, Inc., San Diego, CA). Statistical analyses were performed using the unpaired Student’s t-test. 

Reverse transport (release) was analyzed, as described [10]. Cells loaded with [^3^H]substrate were incubated with or without the drug under investigation at 37°C for 2 min, and separated from the incubation solution. The radioactivity retained in the cells and also in the separated solution was measured by liquid scintillation counting. Statistical tests were performed using an analysis of variance (Kruskal-Wallis test) with pairwise comparisons using Dunn’s multiple comparison test.

### Cell Toxicity Assay

The amount of lactate dehydrogenase (LDH) released into the culture medium was measured for the evaluation of methylone’s toxicity, as described previously [12] with some modifications. Briefly, cells cultured in 96-well culture plates were washed and incubated without phenol red in α-MEM supplemented with 1% BSA and different concentrations of methylone and/or methamphetamine for 24 h. The amount of LDH released into the medium was measured colorimetrically (Wako, Tokyo, Japan). Statistical analyses were performed using the Kruskal-Wallis test and Dunn’s multiple comparison test.

## RESULTS

### Effects of Methylone on the Uptake of Substrate in CHO Cells Stably Expressing Monoamine and GABA Transporters

**Fig. (1) Effects of methylone on the uptake of substrates in CHO cells stably expressing monoamine and GABA transporters.**
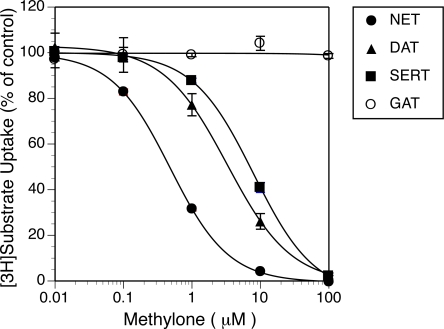
Cells were incubated with [^3^H]substrates at 10 nM in the absence or presence of methylone at various concentrations. Specific uptake was expressed as a percentage of the control, and values represent the mean ± SEM, n=3. Control uptake in the absence of drugs was 4715 ± 182, 4961 ± 170, 13964 ± 1135, and 798 ± 114 dpm/ well for DAT, NET, SERT and GAT, respectively.

The effect of methylone on the transport of monoamines was examined in CHO cells stably expressing the rat monoamine transporters, rDAT, rNET and rSERT, in comparison with that on the mouse GABA transporter, mGAT1. Simultaneous incubation with [^3^H]DA, [^3^H]NE, or [^3^H]5HT and methylone caused a decrease in the uptake of [^3^H]substrate in a concentration-dependent fashion, although the effects differed between transporters in contrast to the effects of methamphetamine (Fig. **1**). The rank order of the transporters in terms of the potency with which they were inhibited by methylone was NET > DAT >> SERT (Fig. **1** and Table **1**). Methylone inhibited SERT more, DAT and NETless, than methamphetamine. However, it had no effect on GAT transport activity at concentrations up to 1 mM (Fig. **1**), while nipecotic acid, an inhibitor of neuronal GABA transporter such as GAT1, inhibited [^3^H]GABA uptake in a concentration-dependent manner (data not shown). 

Next, we analyzed the effect of methylone on uptake kinetically. Table **2** summarizes the effects on [^3^H]substrates in comparison with those of methamphetamine. Methylone increased the K_m_ value without changing the V_max_ for the uptake of [^3^H]NE, indicating competitive inhibition similar to metamphetamine (Table **2**). On the other hand, it showed uncompetitive inhibition of [^3^H]DA and [^3^H]5-HT, tending to decrease the V_max_ while increasing the K_m_, as methamphetamine did. 

### Effects of Methylone and Methamphetamine on the Reverse Transport of [^3^H]substrates 

**Fig. (2). Effects of methylone and methamphetamine on the reverse transport of [3H]substrates through the monoamine transporters.**
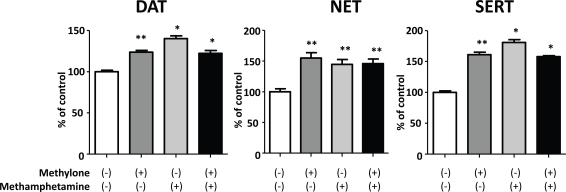
CHO cells stably expressing rDAT, rNET, or rSERT were preloaded with a [^3^H]substrate at 20 nM for 30 min. They were then washed with KRH buffer, and incubated for 2min in fresh medium containing 100 µM methylone and/or 100 µM methamphetamine. Values represent the mean ± SEM, n=8-11, and are expressed as a percentage of the control (without drugs). *P< 0.05, **P< 0.01 vs control.

To further characterize the effects of methylone on the monoamine transporters, we examined its influence on the reverse transport of [^3^H]substrates through DAT, NET and SERT in comparison with methamphetamine, since unlike cocaine, methamphetamine induces the release of monoamines *via* a reversal of transport [13]. Methylone elicited the release of [^3^H]DA, [^3^H]NE and [^3^H]5-HT from the cells expressing rDAT, rNET and rSERT, respectively, similar to methamphetamine (Fig. **2**). In addition, the combination of methylone and methamphetamine did not cause a further increase in the release of [^3^H]substrates. 

### Cytotoxicity Elicited by Methylone and/or Methamphetamine

Initially, we examined the effect of methylone and methamphetamine on cell viability using a MTT-based WST-1 assay previously applied to Foxy [10]. However, we found no changes with methylone and/or methamphetamine at any concentrations tested (data not shown). Therefore, we used a LDH release assay to evaluate methylone’s toxicity.

**Fig. (3). Effect of methylone and methamphetamine on viability of CHO cells stably expressing monoamine and GABA transporters.**
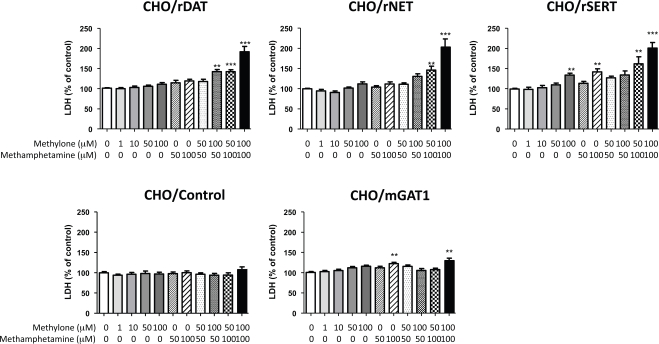
Cells were seeded on 96-well culture plates and cultured for 24 h prior to treatment with the drug under examination. They were then incubated with various concentrations of methylone and/or methamphetamine for 24h, after which the amount of LDH released into the medium was determined. Values are expressed as a percentage of the control (absence of drugs), and represent the mean ± SEM, n=6-16. **P<0.01, ***P<0.001 vs control.

Fig. (**3**) shows the cytotoxicity of methylone and methamphetamine in CHO cells stably expressing rDAT, rNET and rSERT, together with mGAT1 as a control. Methylone did not induce the release of LDH from any cell line except that expressing SERT (Fig. **3**). Furthermore, in combination with methamphetamine it caused a significant increase in the release of LDH in the cells stably expressing the monoamine transporters but not in the control CHO cells or cells expressing GAT (Fig. **3**).

## DISCUSSION

The present study demonstrated that methylone acts as a non-selective inhibitor of the monoamine neurotransmitter transporters DAT, NET and SERT, with a rank order in terms of potencies of NET > DAT >> SERT. Kinetic analyses revealed the characteristics of the inhibition to resemble those by methamphetamine. In addition, methylone induced a reversal of transport similar to methamphetamine. Methamphetamine, like amphetamine, is transported by DAT and NET, and probably SERT [13]. The present study demonstrated that methylone had similar properties to methamphetamine not only in inhibiting the uptake by transporters but also in reversing the direction of transport, suggesting that it too is likely to be a transportable inhibitor. However, methylone itself was cytotoxic only at high concentrations, though in combination with methamphetamine it had a significant effect on cells expressing the monoamine transporters but not the control CHO cells or cells expressing GAT. These results suggest that the transport of methylone through monoamine transporters underlies the cytotoxicity. 

The effectiveness of methylone and methamphetamine in inhibiting the uptake of substrates by monoamine transporters demonstrated here was well consistent with previous findings [5, 6]. Cozzi *et al*. [5] found that methylone inhibited DAT more than NET, while Nagai *et al*. [6] and ourselves observed a more potent effect on NET than DAT. On the other hand, Nagai *et al*. [6] reported that methylone inhibited DAT and SERT equally, while Cozzi *et al*. [5] and ourselves found the effect to be weaker at SERT than DAT. The discrepancy may be due to the different preparations or concentrations of labeled substrates used. 

The concentrations of methylone in the brain at dosages at which the drug is abused, 100 – 200 mg [14], are unknown. Experiments with rats demonstrated the intraperitoneal administration of methylone at 5 mg/kg to be followed by a rapid increase in the plasma concentration ranging from 700 to 1500 ng/mL within 15 or 30 min [11]. These values seem compatible with those for MDMA [7]. Therefore, the present findings suggest that methylone may inhibit monoamine transporters in the CNS at concentrations relevant to its abuse. 

The present study demonstrated cytotoxicity at high concentrations, as assessed from the amount of LDH released in CHO cells. This effect was observed in cells expressing the monoamine transporters, especially those expressing SERT, but not cells expressing mGAT1, suggesting a relationship with the transport of methylone, although the affinity of the transporter for methylone does not explain the potency of methylone’s toxicity. In addition, the present study demonstrated that methylone and methamphetamine combined had a supra-additive effect on the release of LDH in CHO cells expressing monoamine transporters. The cytosolic accumulation of monoamines or methamphetamine may cause oxidative stress, resulting in cell death [15]. Therefore, one may assume that methylone modulates the toxic effects of other monoaminergic agents, such as methamphetamine and 3,4-methylenedioxymethamphetamine (MDMA), through interaction at monoamine transporters. Recently, Shimizu *et al*. [14] reported a case study of a 27-year-old male who took methylone and 5-MeO-MIPT after ingesting a drug powder called pure methylone obtained via the internet, suggesting that substance-related disorders may be complicated by the combined use of psychoactive drugs. According to recent analyses, “ecstacy” and other designer drugs consist of mixtures of MDMA and other substances [16, 17]. Therefore, it is important to note their cytotoxicity when taken simultaneously. Further study is needed to clarify this issue.

In summary, we investigated the effects of methylone on monoamine transporters. The ability of methylone to inhibit transporter function, and damage cells heterologously expressing monoamine transporters, suggests that the transport of methylone underlies its cytotoxicity.

## Figures and Tables

**Table 1:** Effects of Methylone and Methamphetamine on the Uptake of Monoamines in CHO Cells Stably Expressing rat DAT, NET and SERT

Transporter	IC50 (µM)	
	Methylone	Methamphetamine
DAT	2.84 ± 0.36	0.65 ± 0.06
NET	0.48 ± 0.03	0.16 ± 0.00
SERT	8.42 ± 1.01	27.62 ± 2.87

Values represent the mean ± SEM for three experiments each performed in triplicate.

**Table 2:** Changes in the Transport Kinetics Induced by Methylone and Methamphetamine in CHO Cells Stably Expressing the Monoamine Transporters

	K_m_ (µM)	V_max_ (fmol/µg protein/min)
DAT		
Control	1.73 ± 0.55	34.12 ± 7.64
Methylone 5µM	2.33 ± 0.67	26.68 ± 8.70
Methamphetamine 1µM	2.06 ± 0.83	24.18 ± 9.89
NET		
Control	0.25 ± 0.03	3.63 ± 0.31
Methylone 0.5µM	0.58 ± 0.10*	4.54 ± 1.01
Methamphetamine 0.2µM	0.71 ± 0.20	4.69 ± 0.49
SERT		
Control	0.27 ± 0.04	40.68 ± 7.23
Methylone 10µM	1.12 ± 0.25*	36.95 ± 5.68
Methamphetamine 30µM	0.86 ± 0.21*	31.41 ± 4.63

Values represent the mean ± SEM for three experiments each performed in triplicate.

* P<0.05 vs control.
